# The chronic autoimmune thyroiditis quality of life selenium trial (CATALYST): study protocol for a randomized controlled trial

**DOI:** 10.1186/1745-6215-15-115

**Published:** 2014-04-09

**Authors:** Kristian Hillert Winther, Torquil Watt, Jakob Bue Bjørner, Per Cramon, Ulla Feldt-Rasmussen, Christian Gluud, Jeppe Gram, Mogens Groenvold, Laszlo Hegedüs, Nils Knudsen, Åse Krogh Rasmussen, Steen Joop Bonnema

**Affiliations:** 1 Department of Endocrinology and Metabolism, Odense University Hospital Kloevervaenget 4-6, DK-5000 Odense C, Odense, Denmark; 2 Department of Medical Endocrinology, Rigshospitalet, Copenhagen University, Copenhagen, Denmark; 3 National Research Centre for the Working Environment, Copenhagen, Denmark; 4 Institute of Public Health Science, Copenhagen University, Copenhagen, Denmark; 5 Copenhagen Trial Unit, Centre for Clinical Intervention Research, Rigshospitalet, Copenhagen, Denmark; 6 Department of Endocrinology, Hospital of Southwest Denmark, Esbjerg, Denmark; 7 Department of Oncology, Bispebjerg Hospital, Copenhagen, Denmark; 8 Department of Endocrinology, Bispebjerg Hospital, Copenhagen, Denmark

**Keywords:** chronic autoimmune thyroiditis, hypothyroidism, quality of life, selenium supplementation, ThyPRO

## Abstract

**Background:**

Patients with chronic autoimmune thyroiditis have impaired health-related quality of life. The thyroid gland has a high selenium concentration, and specific selenoprotein enzyme families are crucial to immune function, and catalyze thyroid hormone metabolism and redox processes in thyroid cells. Previous randomized controlled trials have found that selenium supplementation decreases thyroid-disease-specific antibody levels. We hypothesize that selenium might be beneficial in the treatment of chronic autoimmune thyroiditis.

**Methods/Design:**

The CATALYST trial is an investigator-initiated randomized, blinded, multicentre clinical trial of selenium supplementation versus placebo in patients with chronic autoimmune thyroiditis. Inclusion criteria: age ≥18 years; serum thyroid peroxidase antibody level ≥100 IU/ml within the previous 12 months; treatment with levothyroxine and written informed consent. Exclusion criteria: previous diagnosis of toxic nodular goitre, Graves’ hyperthyroidism, postpartum thyroiditis, Graves’ orbitopathy; previous antithyroid drug treatment, radioiodine therapy or thyroid surgery; immune-modulatory or other medication affecting thyroid function; pregnancy, planned pregnancy or breastfeeding; allergy towards any intervention or placebo component; intake of selenium supplementation >55 μg/day; inability to read or understand Danish or lack of informed consent. The trial will include 2 × 236 participants. The experimental intervention and control groups will receive 200 μg selenium-enriched yeast or matching placebo tablets daily for 12 months. The experimental supplement will be SelenoPrecise^®^. The primary outcome is thyroid-related quality of life assessed by the Thyroid Patient-Reported Outcome (ThyPRO) questionnaire. Secondary outcomes include serum thyroid peroxidase antibody concentration; serum triiodothyronine/thyroxine ratio; levothyroxine dosage; adverse reactions and serious adverse reactions and events.

**Discussion:**

In this pragmatic trial, participating patients follow their usual treatment at their usual hospitals. In order to collect high-quality data on the clinical course and quality of life, and to minimize missing data, an elaborate trial management system has been designed. 12 months intervention duration was selected in consideration of the primary outcome, thyroid-related quality of life.

**Trial registration:**

ClinicalTrials.gov ID: NCT02013479.

## Background

Chronic autoimmune thyroiditis (AIT), also known as chronic lymphocytic thyroiditis or Hashimoto’s thyroiditis, is a common autoimmune disease, which most often leads to impaired function of the thyroid gland [1]. Its aetiology is based on a complex and poorly understood interaction between genetic susceptibility [2] and a number of environmental triggers [3,4]. In Denmark, the overall yearly incidence of overt hypothyroidism, that is*,* decreased circulating thyroid hormone levels, has been estimated at 47.2/100,000 [5]. AIT has been estimated to account for around 85% of these patients and is 8 to 9 times more frequent in women than in men [5,6]. Thyroid hormone production declines slowly in patients with AIT, with 5% per year progressing to overt hypothyroidism [7]. Standard treatment of AIT is levothyroxine (LT4), based on its long half-life of 8 days and stable generation of triiodothyronine (T3) by deiodination in peripheral tissues [8]. The goal of treatment is to restore euthyroidism and resolve symptoms of hypothyroidism. This is generally accomplished by adjusting the dosage of LT4 to normalize serum thyrotropin (TSH) levels. With rare exceptions, LT4 treatment is lifelong in patients with AIT [9]. According to a comprehensive review from 2006, health-related quality of life (HRQL) is impaired in most patients with benign thyroid disorders. However, the authors emphasized limitations due to lack of a validated and standardized questionnaire for thyroid-related quality of life assessment [10]. Subsequently, we have developed such a thyroid-related quality of life questionnaire, ThyPRO, to overcome the shortcomings and lack of validation of earlier HRQL questionnaires [11-14].

Large-scale community-based studies have demonstrated impaired well-being in euthyroid patients on LT4 treatment for primary hypothyroidism, indicating that restoration of euthyroidism is not the only predictor of good quality of life in patients with AIT [15,16]. In fact, hypothyroidism, even when adequately treated, is associated with increased morbidity and mortality [17-20]. In a cross-sectional study, by investigators from our group, responses to ThyPRO were analyzed in relation to thyroid volume, thyroid function and thyroid autoantibodies. Results suggested that thyroid peroxidase antibody (TPO-Ab) positivity *per se* might be associated with symptomatic distress in patients with AIT [21].

Selenium is a nonmetal mineral, a trace element and an essential micronutrient, incorporated into selenoproteins as the 21st amino-acid, selenocysteine. Selenoproteins have a wide range of effects in, for example, redox homeostasis, immunity, reproduction and thyroid hormone metabolism [22]. The recommended daily selenium intake in Denmark is 50 μg and 40 μg for men and women, respectively, and it is believed that 10% of Danish adults would benefit from increasing their intake [23]. In a recent Danish study, serum selenium concentration was inversely associated with thyroid volume, supporting a role for selenium in the thyroid gland, which indeed has the highest selenium concentration of all tissues [22,24]. A number of selenoproteins are expressed in thyrocytes. The most important of these are the iodothyronine deiodinases (DIO1, DIO2) that catalyze the formation of T3 from T4, and the glutathione peroxidases (GPx1, GPx3, GPx4) that catalyze the removal of peroxides and protect the thyrocytes from oxidative stress [25]. Two randomized clinical trials have shown no effect of selenium supplementation on serum TSH and thyroid hormone concentrations in healthy adults, even in those with moderately deficient selenium status [26,27]. Meanwhile, in the majority of 13 randomized clinical trials, selenium, in various formulations, effectively decreased serum TPO-Ab concentrations in AIT patients [28-40]. Some of the trials also reported other beneficial effects, including improvements in quality of life, although with limited presentation of the data supporting this claim. However, a recent systematic review concluded that evidence to support or discard the efficacy of selenium supplementation in AIT patients is inadequate, and highlights the need of additional randomized placebo-controlled trials to investigate direct clinical outcomes, in order to aid clinical decision making [41].

Only two randomized clinical trials have investigated the effect of selenium supplementation in patients with Graves’ disease, the other major autoimmune thyroid disease. One trial concluded that patients with Graves’ hyperthyroidism who received selenium, in a formulation with multiple antioxidants and in addition to antithyroid drugs (ATDs), attained euthyroidism faster than patients treated with only ATDs [42]. The other trial evaluated the effect of 200 μg selenium selenite daily in 159 euthyroid patients with mild Graves’ orbitopathy, and demonstrated improved disease-specific quality of life and a reduced disease severity, as compared with placebo [43]. We are conducting a trial entitled ‘Selenium supplementation in patients with Graves’ hyperthyroidism (GRASS)’ (ClinicalTrials.gov NCT01611896). The purpose of the GRASS trial is to investigate whether selenium, in addition to the standard treatment with ATDs, in patients with Graves’ hyperthyroidism, will lead to a decrease in antithyroid treatment failure, faster remission and improved quality of life during the first year of treatment. Enrolment of 492 patients into the GRASS study was initiated in December 2012. Patients are randomized to intervention with 200 μg/day of selenium-enriched yeast versus placebo for 24 to 30 months. The CATALYST and GRASS trials are closely connected in terms of study design and will largely be performed by the same group of investigators. Therefore, some sections of the CATALYST protocol are identical to the GRASS protocol [44].

We hypothesize that the addition of selenium to the standard treatment with LT4 in patients with AIT may lead to an improved quality of life and reduce autoimmune disease activity.

## Methods/design

### Objectives

The primary objective is to investigate the effect of 12 months’ intervention with selenium supplementation compared with placebo in patients with AIT, on thyroid-related quality of life, as measured using the ThyPRO questionnaire.

Secondary objectives are to investigate the effect of selenium supplementation compared with placebo on: LT4 dosage; serum T3/T4-ratio; serum TPO-Ab concentration; plasma selenium concentration; and immunological and oxidative stress biomarkers. Monitoring adverse and serious adverse events and reactions constitute additional secondary objectives.

### Design

**Figure 1:**
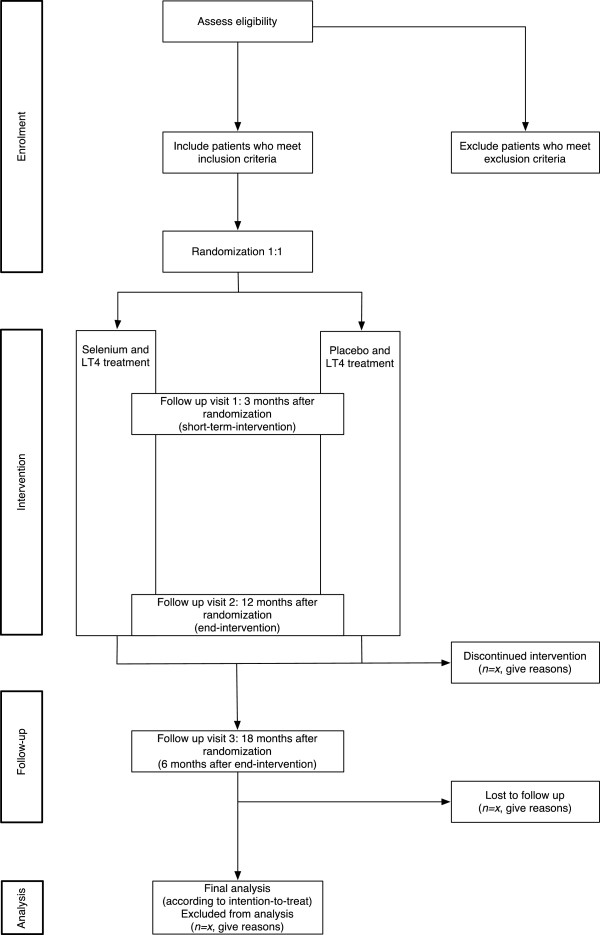
Trial flow chart (adapted from the CONSORT statement 2010).

The CATALYST trial is an investigator-initiated randomized, blinded, multicentre clinical trial of selenium supplementation versus placebo in patients with AIT. The trial has a parallel-group design with 1:1 allocation to the experimental intervention group and the control intervention group (Figure 1). Participants will be recruited from four clinical trial sites in Denmark (Odense University Hospital, Rigshospitalet, Bispebjerg Hospital, and the Hospital of Southwest Denmark). The trial also includes a register-based follow-up during the trial and during a period after completion of the intervention.

### Trial participants

All patients with a diagnosis of AIT who are referred to, or followed-up at, a participating clinical trial site will be considered for participation in this trial. In addition, clinical trial sites may invite participants based on local trial site patient data or advertise in local media or via patient organizations. Patients may enter the CATALYST trial if they comply with the inclusion and exclusion criteria.

#### ***Inclusion criteria***

Age ≥18 years; diagnosis of AIT with serum TPO-Ab ≥ 100 IU/ml measured within the last 12 months; LT4 treatment based on an initial serum TSH ≥ 4.0 mU/l; and written informed consent.

#### ***Exclusion criteria***

Previous diagnosis of toxic nodular goitre, Graves’ hyperthyroidism, postpartum thyroiditis or Graves’ orbitopathy; previous radioiodine therapy, ATD treatment or thyroid surgery; morbidity, rendering the participant unable to process patient-reported outcomes or receive intervention during the trial; immunomodulatory medication; other medication known to affect thyroid function; pregnancy, breastfeeding or planned pregnancy within the next 18 months; allergy towards any component in the selenium or the placebo tablets; intake of selenium supplementation >55 μg/day; inability to read or understand Danish; or lack of informed consent.

### Trial intervention

#### ***Selenium***

In this trial, oral tablets of selenium-enriched yeast will be used. The experimental intervention will be 200 μg selenium per day, corresponding to two 100 μg tablets SelenoPrecise^®^ in a formulation registered as a dietary supplement and produced by Pharma Nord ApS, Vejle, Denmark.

#### ***Placebo***

The control intervention will be two placebo tablets per day as inactive spray-dried baker’s yeast, comprising 250 mg yeast placebo, 80 mg cellulose, 65 mg dicalcium phosphate, and ≤5 mg other inactive ingredients. Tablets will be identical to the experimental intervention as regards size, appearance, taste, smell and solubility and will be produced by Pharma Nord ApS, Vejle, Denmark.

#### ***The trial data management system (TDMS)***

Since the design will involve limited interaction between participants and trial personnel, a considerable part of the data collection, trial conduct, trial surveillance and timing, is handled by a trial data management system TDMS. This system, named PROgmatic, is described in detail in an independent publication [45]. It consists of a patient-survey interface, a trial-personnel interface, a system-integration interface and a program ‘motor’. The system features include secure web-based data entry; electronic case report forms (eCRFs); central participant registration and randomization; automated emails linking to electronic patient-reported outcomes; automated reminders to participants; automated notifications to trial personnel regarding booking of trial visits, safety and compliance alerts and monitoring of trial progress. It will be used for collecting outcome data, adverse events, and other study-relevant information; for timing of trial events, that is, time for patient-reported outcomes and trial visits; for identification of need for action (for example, contact to a participant); and for delivery of output to personnel or participants (for example, email notifications). In this way, the eCRFs are created by integration of patient-input, trial-personnel input and collection of electronic data regarding biochemical analyses (that is, blood-tests taken as part of the usual routine). All activities are automatically logged. The TDMS complies with the security policies of the participating hospitals and the Act on Processing of Personal Data, and is approved by the Danish Data Protection Agency.

#### ***Randomization***

Randomization will be performed centrally by a consultant at the SAS Institute A/S (Copenhagen, Denmark). The allocation sequences will be computer-generated with varying block size, and will be kept unknown to the investigators. Randomization will be stratified by clinical trial site and LT4 treatment duration (<3 months or ≥3 months) and the allocation ratio of intervention and control will be 1:1.

#### ***Informed consent procedure***

Potential participants include all patients with suspected AIT who receive LT4 and are identified at referral or visits to the outpatient clinics or via advertising, as previously described. When AIT is confirmed (serum TPO-Ab ≥ 100 IU/ml, measured within the last 12 months), the patient is invited to an information visit. This visit involves obtaining medical history and written informed consent, blood and urine sampling, and information about trial contact as well as randomization.

#### ***Concomitant medication or treatment***

The trial interventions are given in addition to conventional LT4 treatment. Treatment of the hypothyroidism *per se* will take place at participating clinical trial sites, with LT4 treatment at all clinical trial sites aiming for a serum TSH level within reference ranges, as recommended in local clinical guidelines. Patients are normally followed with blood sampling every 1 to 3 months to adjust LT4 dosage according to TSH levels, as well as visits in the outpatient clinic every 3 to 6 months. Participants in the CATALYST trial will be followed with blood sampling during the trial period, as stated in local clinical guidelines. Participants are advised not to take selenium supplementation in addition to the intervention. A dose of selenium common in multivitamin tablets is, however, allowed and participants are instructed and informed about this upon trial entry, and in the written participant information. The participants’ consumption of additional selenium supplements will be monitored during the trial at specific time points.

#### ***Monitoring participant adherence to intervention***

During the trial, participants self-report their adherence to the intervention at the trial visits (3 months and 12 months after randomization) by stating the number of days a week they take their tablets. Participants will also be instructed to bring remaining tablets and empty containers to the 12 month visit, where they will be counted and registered by an investigator or trial nurse.

#### ***Discontinuation***

A participant who no longer wishes to participate in the trial can withdraw informed consent at any time without need of further explanation, with no consequence on the participant’s further treatment. To conduct intention-to-treat analyses, with as little ‘missing data’ as possible, the investigator may ask the participant which aspects of the trial he or she wishes to withdraw from. This could involve the trial intervention, participation in the remaining follow-up assessments, or use of already collected data in the data analyses. The investigators shall discontinue a participant from the trial intervention at any time, if the participant complies with any of the exclusion criteria during the intervention period or experiences intolerable adverse reactions. In both cases, the investigator or the treating physician shall, if possible, encourage the participant to continue with follow-up assessment and allow the use of already collected data in the analyses.

#### ***Blinding***

Blinding will be maintained for all parties in the trial, throughout all aspects of the trial. Statistical analyses will be performed with the blinding intact, for example, the experimental intervention and control intervention group will be randomly coded as ‘A’ and ‘B’. Two conclusions will be drawn; one assuming that ‘A’ is the experimental intervention group and ‘B’ is the control intervention group, and one assuming the opposite. Only after this will the blinding be broken.

### Safety

The selected dosage and the exclusion criterion regarding additional selenium supplementation intake ensure that participants have a total selenium intake of less than 300 μg per day, as recommended in current European guidelines on tolerable upper intake level [46]. It has previously been concluded that SelenoPrecise^®^ is adequately characterized, of reproducible quality, and that there is no evidence of toxicity even at intake levels far above 300 μg per day [47]. In a randomized clinical trial (UK PRECISE) with 501 volunteer participants from general practice, mean plasma selenium increased from 88.1 μg/l to 188.1 μg/l following 6 months supplementation with 200 μg/day SelenoPrecise [48]. While no serious adverse events occurred, 12 adverse events were reported, primarily stomach or abdominal discomfort, which were equally distributed between the selenium and placebo groups [48]. The tablets used were identical to the planned experimental intervention agent in CATALYST, and CATALYST participants are expected to be very similar to the UK PRECISE participants, with regard to baseline selenium status.

The safety profile of the dietary supplement used in the CATALYST trial is based on the summary of product characteristics (SPC) for the drug formulation of SelenoPrecise^®^ from the Danish Medicines Agency. Common adverse reactions (between 1/10 and 1/100) are complaints from the gastrointestinal system, while uncommon (between 1/100 and 1/1,000) and rare (between 1/1,000 and 1/10,000) adverse reactions are mainly of a dermatological nature.

### Outcomes

Data will be collected for outcome assessment at six specified times during the trial (Table 1).

**Table 1:** Timing of data collection

**Variable**	**Inclusion baseline**	**Follow-up 6 weeks ± 1 week**	**Follow-up 3 months ± 2 weeks**	**Follow-up 6 months ± 2 weeks**	**Follow-up 12 months ± 2 weeks**	**Follow-up 18 months ± 2 weeks**
Visit	*x*		*x*		*x*	*x*
Weight	*x*_*v*_		*x*_*v*_		*x*_*v*_	*x*_*v*_
Medical treatment	*x*_*v*_		*x*_*v*_		*x*_*v*_	*x*_*v*_
ThyPRO	*x*_*r*_	*x*_*r*_	*x*_*r*_	*x*_*r*_	*x*_*r*_	*x*_*r*_
TSH, FT4, FT3	*x*_*s*_		*x*_*s*_		*x*_*s*_	*x*_*s*_
TPO-Ab	*x*_*s*_		*x*_*s*_		*x*_*s*_	*x*_*s,*_
LT4 dosage	*x*_*v*_		*x*_*v*_		*x*_*v*_	*x*_*v*_
Immunological and oxidative stress biomarkers	*x*_*s*_		*x*_*s*_		*x*_*s*_	*x*_*s*_
Selenium	*x*_*s*_		*x*_*s*_		*x*_*s*_	*x*_*s*_
Creatinine/iodine ratio in spot urine	*x*_*s*_					
Tablet count			*x*_*v*_		*x*_*v*_	
Consumption of additional selenium	*x*_*v*_		*x*_*v*_		*x*_*v*_	*x*_*v*_
Adverse reactions			*x*_*v*_		*x*_*v*_	*x*_*v*_
Serious adverse reactions and events (SARs, SUSARs and SAEs)						*x*_*p*_

All assessments must be made at the time points specified above. If not possible at the specified time, the assessment shall still be conducted, and the time of assessment shall be noted in the specified electronic case report form (eCRF). *x*_*v*_, analyzed via data obtained in eCRFs at trial visits, administered through the trial data management system (TDMS); *x*_*r*_, analyzed via patient-reported outcomes, administered through the TDMS; *x*_*s*_, analyzed in blood or urine samples obtained at trial visits; *x*_*p*_, analyzed via public national registries at the end of the trial.

#### ***Primary outcome***

Improved thyroid-related quality of life during the 12 months intervention after randomization, as measured by one composite score from the ThyPRO questionnaire [49].

#### ***Secondary outcomes***

Secondary outcome assessment is specified in Table 1. Forkhead Box P3 mRNA will be measured as an immunological biomarker, and urinary 8-oxo-2'-deoxyguanosine and 8-oxoguanine will be measured as oxidative stress biomarkers.

#### ***Assessment of adverse reactions and events***

The latest version of the SPC shall at all times be used for the assessment of adverse reactions (ARs), which will be reported as a secondary trial outcome. Participants are questioned about ARs according to Table 1. In addition, participants are instructed to contact their trial contact person if they experience symptoms suggestive of ARs.

In the assessment and reporting of serious adverse reactions (SARs), serious unexpected serious adverse reaction (SUSARs) and serious adverse events (SAEs), data on hospital admissions and mortality will be obtained through national registries at the end of the trial. Also, participants are informed and instructed to contact their trial contact person if they are admitted to a hospital for selenium intoxication, experience a clinical picture indicative of selenium intoxication, or experience a clinical picture that is unexpected but suspected to be related to selenium intoxication. When a possible serious event (SAE, SAR or SUSAR) is identified, details will be sought from the patient’s medical record and through direct contact with the patient. Any SAE, SAR or SUSAR will be reported as an outcome measure.

### Research biobank

A central research biobank, of buffy coat, serum, plasma and urine samples, will be established for analyses of biochemical markers specified in the outcome section and for future use. Future sample analyses include genome-wide association studies (regarding predictors of experimental intervention effect, or other predictors of autoimmunity) as may be specified in forthcoming protocols. Participants are informed orally and in writing, and will consent to the withdrawal and storing of biological material in the CATALYST trial. For this purpose, tubes of blood and urine will be collected at each of the four trial visits. Trained trial personnel will perform blood sampling; the risks involved with blood sampling are no more than with standard blood sampling.

### Monitoring

The trial will be monitored according to the International Committee of Harmonisation guidelines for good clinical research practice (ICH-GCP) by internal monitoring [50].

### Statistical analyses

#### ***Primary outcome: sample size estimation***

The primary outcome is thyroid-related quality of life during 12 months’ intervention, as measured by a composite score from the ThyPRO questionnaire. Sample size estimation is based on this outcome.

The trial should be sufficiently powered to identify a difference between the intervention and the control group of four points on the 0 to 100 ThyPRO composite scale, corresponding to a small to moderate effect. In previously obtained data, the standard deviation of ThyPRO-scores (sigma level) was 20 points [14]. With a correlation between observations on the same participant of 0.50, and a power of 80% and a type I error probability (two-sided α level) of 0.05, a sample size of 236 experimental participants and 236 control participants is required. The sample size estimate is based on a design with five repeated measurements having a compound symmetry covariance structure [51].

#### ***Secondary outcomes: power estimation***

Power calculations are based on serum TPO-Ab concentrations and LT4 dosage. Standard deviations used for power calculations were extracted from unpublished data in a study population of approximately 200 AIT patients from Copenhagen and Odense University Hospitals receiving LT4 treatment [14].

#### ***Serum TPO-Ab concentration***

While a previous study has linked TPO-Ab positivity in hypothyroid patients to impaired HRQL [20], we have not found conclusive evidence in the literature that the level of serum TPO-Ab among participants in this trial, that is, AIT patients with serum TPO-Ab concentration ≥100 IU/ml, has clinical implications. The power estimate is based on a design with three repeated measurements having a compound symmetry covariance structure, with a standard deviation (σ level) of 535 IU/ml (unpublished data) [14], a correlation between observations on the same participant of 0.50, and a type I error probability (two-sided α level) of 0.05. Under these circumstances, a difference in serum TPO-Ab levels of 138 IU/ml between the experimental and control group can be identified with 80% power. We find the power of the trial to assess effect on serum TPO-Ab concentrations acceptable.

#### ***LT4 dosage***

In this trial, a difference in LT4 dosage of 25 μg/day between the experimental and control group following intervention should be detected, since 25 μg/day is a minimal dosage adjustment readily made by clinicians, when patients are treated with the most common LT4 formulation (Eltroxin) on the Danish market. The power estimate is based on a design with five repeated measurements having a compound symmetry covariance structure, with a standard deviation (σ level) of 81 μg/day (unpublished data) [14], a correlation between observations on the same participant of 0.50, and a type I error probability (two-sided α level) of 0.05. Under these circumstances, the probability (power) of finding a true difference in LT4 dosage of 25 μg/day between the experimental and control group following intervention is 92%, which we find acceptable.

#### ***Expected participant recruitment***

The patient uptake areas for the participating clinical trial sites comprise a total of 910,000 persons (Rigshospitalet: 70,000; Odense University Hospital: 350,000; Bispebjerg Hospital: 270,000; Hospital of Southwest Denmark (Esbjerg): 220,000).

Previous studies report an incidence of 40 per 100,000 per year of spontaneous (presumed autoimmune) hypothyroidism after the implementation of iodine fortification in Denmark [5,6]. This corresponds to 364 patients per year in the given uptake area. A considerable proportion of hypothyroid patients are treated in the primary care sector, and will neither be referred to nor followed at a participating clinical trial site. Assuming an inclusion of 30% of the annual incident patients, we will be able to include 109 participants per year or nine participants per month. Half of the 472 participants will be included from the incident (LT4 treatment <3 months) group, and this is expected to take 24 to 26 months. In addition to this, established patients (LT4 treatment ≥ 3 months) with autoimmune thyroiditis are eligible for inclusion. The prevalence of diagnosed and treated hypothyroidism was 1% in a previous iodine fortification program (DanThyr) with a population of 4,073 men and women. 83% of hypothyroid patients had serum-TPO-Ab above 200 IU/ml [5]. An estimated AIT prevalence of 1% among adults in our patient uptake area will be approximately 10,000 individuals. We expect to recruit 236 participants from the prevalent patient group within the 24 to 26 month recruitment period of the incident patients.

#### ***Data analyses***

Analyses will be performed using SAS (SAS Institute Inc. ©, Cary, NC, USA), version 9.3 or later. All analyses will be intention-to-treat analyses performed blinded with the two intervention groups concealed as, for example, ‘A’ and ‘B’. Significance tests will be at the 5% level and two-sided. Table 2 shows, for each outcome, its priority, when it will be measured, the mathematical type of measure, and the analytical procedure to be used when analyzing the outcome values. The analytical procedures and handling of missing values are described next.

**Table 2:** Outcome measures

**Outcome (priority)**	**Times of measurements**	**Mathematical type of quantity (analytical category)**
ThyPRO composite scale score (P)	Time sequence of six measurements^a^	Numerical (1 + 2)
ThyPRO hypothyroid symptoms (S)	Time sequence of six measurements^a^	Numerical (1 + 2)
ThyPRO goitre symptoms (S)	Time sequence of six measurements^a^	Numerical (1 + 2)
LT4 dosage (S)	Time sequence of four measurements^b^	Numerical (1 + 2)
Serum TPO-Ab (S)	Time sequence of four measurements^b^	Numerical (1 + 2)
Serum FT3/FT4 Ratio (S)	Time sequence of four measurements^b^	Numerical (1 + 2)
Plasma selenium (S)	Time sequence of four measurements^b^	Numerical (1 + 2)
Biomarkers of immunology and oxidative stress (S)	Time sequence of four measurements^b^	Numerical (1 + 2)
Adverse reactions (S)	End of trial	Rate (3)
Serious adverse events (S)	End of trial	Rate (3)

Outcomes with priorities, times of measurement and mathematical types of quantity with analytical categories. P, primary outcome measure; S, secondary outcome measure. Analytical categories 1 to 3 are defined in the main text. ^a^, includes five measurements relative to the reference time (6 weeks, 3 months, 6 months, 12 months and 18 months after randomization); ^b^, includes three measurements relative to the reference time (3 months, 12 months and 18 months after randomization).

#### ***Analytical procedures***

Depending on the specific analytical category of each outcome measure, one or two of three types of regression analyses will be applied (Table 2).

#### ***Analytical categories***

*Type 1* includes a continuous outcome measure (*y*) measured 3 to 6 times at 6 weeks, 3, 6, 12 or 18 months after randomization. It is viewed as a linear function of time (*t*). The model is:

*y* = *a* + b*I + c*t + d*I*t

Depending on the mean value structure in the two groups, this model may be enhanced to include a second- or third-degree polynomial. *I* is the intervention indicator, and *a*, *b*, *c* and *d* are the coefficients of the regression equation. A mixed model with repeated measures will be used. An unstructured covariance matrix will be used. In the case of lack of convergence, the following covariance structures will be tried in the specified order: the spatial power law and the compound symmetric.

*Type 2* includes a single continuous outcome measure. The general linear univariate model will be used. As a sensitivity analysis, a nonparametric test (Mann Whitney) will be conducted and the result discussed.

*Type 3* includes a count of events within a specified period of counting as outcome measure. The generalized linear model will be used with the Poisson distribution, link = log and log (period) as offset. As a sensitivity analysis a nonparametric test (Mann Whitney) is conducted to compare the distributions of rates between the groups and the result discussed.

#### ***Missing values***

If the distributions of the primary outcome measure differ significantly between the two intervention groups, multiple imputations (MI) will be used to replace missing data with substituted values using SAS version 9.3 or later. In the MI analysis, the model variables and additional variables significantly related to the variables with missing values of these variables will be used. Ten imputed data sets will be produced.

### Ethical considerations

As specified by the Danish Medicines Agency, the CATALYST trial is not subject under the Danish Medicines Act (law 1180 by 12/12/2005, §88, section 1). The CATALYST trial obeys the rules and regulations set down by The Biomedical Research Ethics Committees, Danish Data Protection Agency, and the health authorities. The CATALYST trial will be conducted in accordance with the Declaration of Helsinki. The trial has been approved by the Regional Scientific Ethical Committees for Southern Denmark (project ID: S-20130123).

## Discussion

The data available from previous trials warrant further investigation, since a benefit from selenium supplementation is still unproven in patients with AIT. In previous trials of intervention with selenium in patients with AIT, effect on serum TPO-Ab concentration has been the primary outcome. Intervention durations of 3, 6, 9, and 12 months using different selenium formulations have shown positive effects on serum TPO-Ab concentrations. This trial will be the largest yet, with 472 participants, and the first to measure changes in quality of life, by ThyPRO, as the primary outcome. The sample size estimate does not take dropouts or off-trial use of selenium supplements into account. Plasma selenium concentrations will be measured to assess selenium intake during the trial. Importantly, utilizing the ThyPRO questionnaire will enable us to determine how selenium supplementation may affect specific aspects of thyroid-related quality of life in AIT patients. In assessing quality of life, the effect of levothyroxine alone will be taken into account by adjusting for TSH levels. Choosing a 12-month intervention period allows for a time sequence of five measurements. The 6-month postintervention follow-up period was added to assess sustained effects. This trial will also look into selenium’s mechanisms of action in AIT, by measuring biomarkers of immunological activity, and will be the first to evaluate whether selenium supplementation affects LT4 dosage. Dosage reduction is of clinical interest and discontinuation of LT4 would be of direct clinical relevance. An effect of selenium supplementation on LT4 dosage is conceivable, owing to upregulation of selenoproteins involved in thyroid hormone synthesis or thyrocyte protection.

Participants will be stratified for LT4 treatment duration of more or less than three months, corresponding to newly diagnosed and established patients, respectively. Participants in the newly diagnosed patient group will respond to LT4 treatment *per se*, in terms of restoration of euthyroidism, while the established patient group will not experience major fluctuation in the thyroid hormone levels during the study period. The stratification also allows an investigation of whether the newly diagnosed and established patient groups respond differently to selenium supplementation. This investigation will, however, be strictly exploratory, as it is likely to be underpowered.

It is of major importance to the CATALYST trial’s initiators that the results will be as directly applicable to daily clinical practice as possible. Therefore, it will be conducted as a pragmatic trial [52], with participating patients following their usual treatment at their usual hospitals in the care of whichever physician is involved. At the same time, it is a priority to collect high-quality data with respect to HRQL, since this is the primary outcome of the trial.

The intent of conducting a pragmatic trial, while still measuring HRQL meticulously, presents a challenge. Considerable effort has therefore been directed at designing a trial data management system that can handle distance from participants and maintain close monitoring [45]. The system initiates and keeps track of patient-reported outcomes and identifies a need for trial personnel input and action. The system also identifies the site of the information provider and LT4 treatment duration of new participants, in order to deliver randomization numbers. Meticulous follow-up of missing responses to the HRQL measurements is incorporated into the system, to minimize the well-known problem with missing HRQL data in clinical trials.

The intent to comply with the ICH-GCP guidelines is also a challenge when conducting a pragmatic trial. In the CATALYST trial, the experimental intervention is a dietary supplement rather than a drug. The need to monitor adverse reactions and events is addressed by a combination of thorough instruction of the participants to contact their trial person in case of symptoms indicative of adverse reactions or events, surveillance of patients’ responses to prompts through the trial data management system and integration with national databases regarding hospitalizations.

## Trial status

The study protocol is approved by the Regional Scientific Ethical Committees for Southern Denmark (project ID: S-20130123) and the first patient visit is scheduled for April 2014. The schedule is specified in Table 3.

**Table 3:** Time schedule

**Time**	**Task**
July 2012 to March 2014	Preparation and approval of trial protocol (Regional Scientific Ethical Committees for Southern Denmark and Danish Data Protection Agency) and trial registration (ClinicalTrials.gov)
April 2014	First patient, first visit
August 2016	Last patient, first visit
February 2018	Last patient, last visit
Spring 2018	Analysis of biological samples and data. Preparation of manuscripts

## Abbreviations

AIT: autoimmune thyroiditis; AR: adverse reaction; ATD: antithyroid drug; DIO: iodothyronine deiodinase; eCRF: electronic case report form; FT3: free triiodothyronine; FT4: free thyroxine; GCP: good clinical practice; GPx: glutathione peroxidase; HRQL: health-related quality of life; ICH-GCP: International Conference on Harmonisation guidelines for good clinical research practice; LT4: levothyroxine; MI: multiple imputations; PRO: patient-reported outcome; SAE: serious adverse event; SAR: serious adverse reaction; SPC: summary of product characteristics; SUSAR: suspected unexpected serious adverse reaction; T3: triiodothyronine; T4: thyroxine; TDMS: trial data management system; ThyPRO: Thyroid Patient-Reported Outcome (thyroid-related quality of life questionnaire); TPO-Ab: thyroid peroxidase antibody; TSH: thyroid stimulating hormone (thyrotropin).

## Competing interests

The authors declare that they have no competing interests.

## Authors’ contributions

KHW: conception and design of the trial, writing and final approval of the manuscript. TW: conception and design of the trial, data collection and analysis, critical revision and approval of final manuscript. JBB and MG: data analysis and interpretation, critical revision and approval of final manuscript. PC, UFR, CG, JG, LH, NK, ÅKR and SJB: conception and design of trial, critical revision and approval of the final manuscript. All authors read and approved the final manuscript.
